# Prediction of Extrathyroidal Extension Using Ultrasonography and Computed Tomography

**DOI:** 10.1155/2014/351058

**Published:** 2014-11-27

**Authors:** Doh Young Lee, Tack-Kyun Kwon, Myung-Whun Sung, Kwang Hyun Kim, J. Hun Hah

**Affiliations:** Department of Otorhinolaryngology-Head and Neck Surgery, Seoul National University Hospital, Seoul National University College of Medicine, 101 Daehak-Ro, Jongno-Gu, Seoul 110-744, Republic of Korea

## Abstract

*Objectives*. The aim of the present study was to evaluate the value of high-resolution ultrasound (US) and computed tomography (CT) scan for preoperative prediction of the extrathyroidal extension (ETE). *Methods*. We analyzed the medical records of 377 patients with papillary thyroid carcinoma (PTC) with preoperative US and CT scan to calculate the sensitivity, specificity, and positive and negative predictive values of characteristics imaging features (such as contact and disruption of thyroid capsule) for the presence of ETE in postoperative pathologic examination. We also evaluated the diagnostic power for several combinations of US and CT findings. *Results*. ETE was present in 174 (46.2%) based on pathologic reports. The frequency of ETE was greater in the patients with greater degrees of tumor contact and disruption of capsule, as revealed by both US and CT scans (positive predictive value of 72.2% and 81.8%, resp.). Considering positive predictive values and AUC of US and CT categories, separately or combined, a combination of US and CT findings was most accurate for predicting ETE (83.0%, 0.744). *Conclusions*. This study suggests that ETE can be predicted most accurately by a combination of categories based on the findings of US and CT scans.

## 1. Introduction

Over the past decades, numerous studies have identified risk factors for patients with papillary thyroid cancer (PTC) [1, 2]. Among the suggested risk factors, extrathyroidal extension (ETE) was repeatedly found to be one of the most important prognostic factors that affect recurrence and survival. For example, the 15-year survival rate of the patients with PTC was found to be significantly lower in patients with ETE than in those without ETE [3, 4]. Therefore, ETE is regarded as a powerful indicator that can affect the recommended surgical extent and the need of postoperative adjuvant therapy. However, the presence of ETE was mainly determined on postoperative pathologic examination. ETE is difficult to be ascertained preoperatively or even intraoperatively, especially when there is no gross invasion into the adjacent structures, in contrast to the more easily evaluated prognostic factors including age, sex, and tumor size.

It is advantageous for surgeons and patients to know the possibility of ETE before surgery, in order to decide the extent of the surgery (lobectomy/total thyroidectomy or central neck dissection). And one of the critical determinants for postoperative radioactive iodine (RAI) treatment is the presence of ETE. Therefore, it is imperative to predict the presence of ETE preoperatively. We considered that modern imaging modalities, such as ultrasonography (US) and/or computed tomography (CT) scan, could be used to evaluate the possibility of ETE preoperatively. Notably, Kim et al. suggested that tumors that were found by US to be attached to the thyroid capsule had a greater risk of ETE than those completely surrounded by thyroid parenchyma [5]. Furthermore, another study suggested that the degree of contact between tumor and the adjacent capsule was a predictor for ETE [6]. However, although imaging quality and techniques of US of the thyroid gland has greatly improved, especially detecting small nodules, US only still cannot predict the all ETE due to the high rate of false positive and negative findings [4].

To date, there have been relatively few studies that assessed the value of CT scan in predicting ETE. The aim of this study was to evaluate the feasibility of high resolution US and CT scans in predicting ETE and the predictive power of the combination of these two modalities.

## 2. Materials and Methods

All protocols and aspects of experimental design were approved by the Institutional Review Board (IRB) of the Seoul National University College of Medicine/Seoul National University Hospital. A retrospective chart review was performed of the medical records of patients undergoing total thyroidectomy (with or without neck dissection) in the Department of Otolaryngology-Head and Neck Surgery, Seoul National University Hospital, between January 1, 2006, and December 31, 2012. Patients with diagnoses other than PTC were excluded, as were those with mixed tumor and those with missing data of US or CT scans.

**Figure 1:**
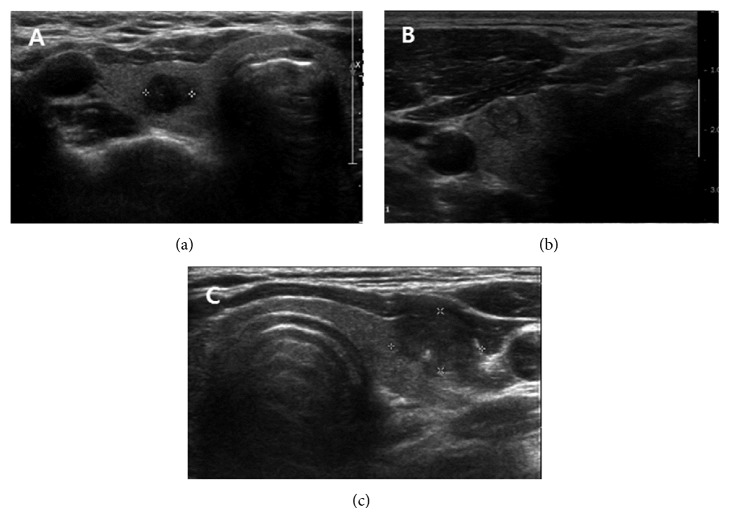
Grouping of thyroid tumor according to ultrasonographic findings. (a) Group 1: the tumor is completely enveloped by thyroid parenchyma, (b) Group 2: the tumor is attached to the thyroid capsule without definite destruction of the capsule, (c) Group 3: the tumor destroys the thyroid capsule and the capsule shadow is disconnected.

Parameters investigated were patient's age, gender, surgical procedure performed, preoperative US and CT findings, and pathologic reports. For US, the thyroid nodule was evaluated by the standard methodology from published reports, including size, composition, echogenicity of the solid tissue, orientation, shape, margin, and calcification [4, 7–11]. We categorize the US tumor characteristics in three groups: Group 1, a tumor which was completely enveloped by thyroid parenchyma; Group 2, a tumor which was attached to the thyroid capsule without definite destruction of the capsule; and Group 3, a tumor attached to the thyroid capsule with loss of capsule shadow (Figure 1).

**Figure 2:**
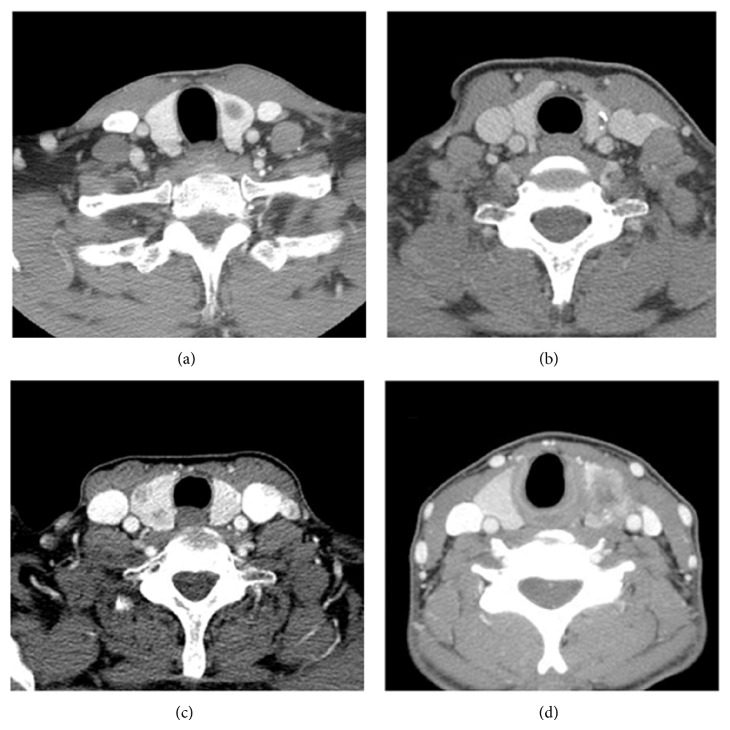
Grouping of thyroid tumor according to computed tomographic findings. (a) Group A: the tumor is completely enveloped by thyroid parenchyma, (b) Group B: the percentage of the perimeter of the tumor that contacted the thyroid capsule is less than 25%, (c) Group C: the percentage of the perimeter of the tumor that contacted the thyroid capsule is 25–50% contact, (d) Group D: the percentage of the perimeter of the tumor that contacted the thyroid capsule is more than 50%.

All CT scans were performed with contrast medium. For CT scan, the characteristics of the tumor were categorized into the following four groups by evaluating all axial, coronal, and sagittal images: Group A, a tumor which was completely enveloped by thyroid parenchyma; Group B, a tumor in which the percentage of the tumor perimeter in contact with the thyroid capsule was 1–25%; Group C, a tumor in which the contact with the capsule was 25–50%; and Group D, a tumor in which the contact with the capsule was >50% (Figure 2). Pathologic report was uniformly categorized into laterality, multicentricity, tumor size, TNM staging, gross and/or microscopic ETE, and the presence of nodal metastasis.

Statistical analysis was performed using the software package SPSS for Windows version 20.0 (SPSS Inc., Chicago, Il). Chi-square tests were used to evaluate the statistical significance of association of the US and CT findings, such as contact and disruption of the capsule with ETE. Student's *t*-test was used to evaluate the statistical significance of differences in mean values of continuous variables. Indicators of predictive performance, including sensitivity, specificity, positive predictive value (PPV), and negative predictive value (NPV), were calculated for the categories of US and CT findings separately or combined. A receiver-operating-characteristic (ROC) curve analysis was performed to evaluate the accuracy of the US and CT findings in predicting the ETE. A *P* value <0.05 was considered statistically significant.

## 3. Results

Table 1 shows demographic data of all patients with or without ETE. The presence or absence of ETE was based on the postoperative pathologic report. Of 377 patients with PTC, ETE was identified in 174 patients (46.2%). Mean age was not different between the two groups. The mean tumor size in patients with ETE, 1.19 cm (±0.36), was significantly larger than that of tumors in patients without ETE, 0.68 cm (±0.23), (*P* = 0.001). Lymph node metastasis was found in 179 (47.5%) patients, which was significantly higher in the patients with ETE than without ETE (*P* < 0.001). The frequency of ETE showed significant difference among 3 groups in US (Group 1; 19.2%, Group 2; 64.7%, Group 3; 88.1%; *P* < 0.001). Similarly, on the CT scan, the frequency of ETE in the pathologic examination was found to increase as the degree of contact between the tumor and the adjacent thyroid capsule, (the frequency of ETE in Group A was 27.8%; Group B, 37.5%; Group C, 50%; and Group D, 81.8%; *P* < 0.001).

Indicators of the diagnostic values of US and CT findings for predicting the ETE are shown in Tables 2 and 3. The PPV was the highest in tumor with >50% contact with the adjacent capsule on CT scan (81.8%) and AUC was the highest when the cutoff criteria was disruption of capsule on US (0.647) (Table 2). When combining two modalities, tumors with disruption of the capsule by US and with >25% contact with capsule by CT scan achieved the highest AUC for ETE (0.744) (Table 3).

Table 4 shows the positive PPV of combining US and CT according to the size of thyroid nodule. When the tumor is less than 1 cm, the combinations of Group 2 on US with Group D on CT scan and Group 3 with Groups B, C, and D showed the PPV over 50%. When the tumor is larger than 1 cm, the combination of Group 2 or Group 3 with Groups B, C, and D shows the PPV over 50%.

## 4. Discussion

To our knowledge, this is the first study that evaluated the diagnostic performance of combined US and CT scan for PTC. From our data, the combination of both imaging modalities showed the highest diagnostic performance. US is the first modality that is used to evaluate the thyroid nodules because it provides greater spatial resolution than CT and affords improved evaluation of nodule architecture and consistency. This information includes the presence of additional coexisting small nodules, which were seen in 93 of 230 patients (40.4%) in our series.

In terms of ETE, only a few previous studies investigated preoperative US findings in relation to predicting ETE. Ishigaki et al. reported that, when cases in which the tumors were protruding into the surrounding tissues were defined as extrathyroid extension in US, the sensitivity, specificity, and accuracy rates were 62.9%, 97.6%, and 81.8%, respectively [12]. Kwak et al. assessed PTC less than 1 cm on the basis of the degree of contact of the tumors with the capsule and suggested that more than 25% contact with the capsule is the most accurate measurement to predict ETE (sensitivity 65.2%, specificity 81.8%, PPV 70.7%) [6]. Diagnostic performance of US in our data corresponded well with these previous studies that evaluate the correlation preoperative ultrasonographic ETE and postoperative findings.

Detection of micropapillary thyroid cancer is rapidly improving due to advances in imaging techniques. With its early detection and low-grade of malignancy, PTC usually has very low mortality and recurrence rates, about 5% and 15%, respectively [13]. However, when there is ETE, even with microscopic extension, the 10-year-survival rate was found to be lower (88%) than that without ETE [14]. Various studies demonstrated that the high-risk group of patients with PTC, which includes those with ETE, had significantly lower survival than the low-risk group of patients. Therefore, the appropriate treatment of tumors with ETE is relevant to lowering the recurrence and mortality rates. In that context, elective bilateral central neck dissection may be considered in unilateral PTC with ETE, because the presence of ETE is closely correlated with regional lymph node metastasis or recurrence [15].

**Figure 3:**
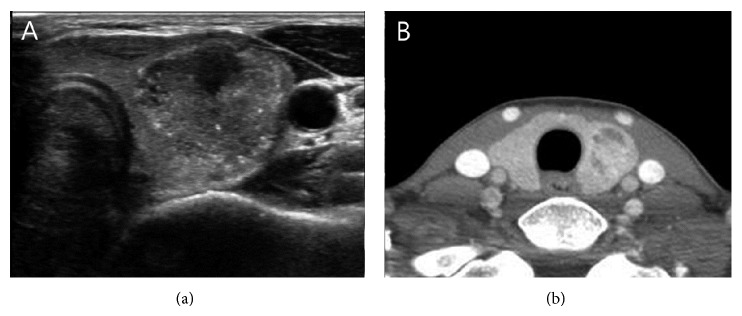
44-year-old woman with papillary thyroid carcinoma without extrathyroidal extension. On contrast to the US finding which shows the tumor closely attached to the capsule, CT findings revealed the complete parenchymal envelop around the tumor.

Although US usually provides critical information in managing the patients with PTC, the US is a subjective and examiner-dependent inconsistent study and shows only 2-dimensional images. Regarding ETE, as observed in previous studies and our data, diagnostic performance is not improved despite technical support and development of US had increased. To overcome these disadvantages, US using shear wave elastography or magnetic resonance imaging has been tried [16, 17]. However, several techniques using US still cannot perfectly assess the ETE preoperatively. Our data showed that the combination of two imaging methods might increase PPV of ETE. CT scan can overcome the disadvantages of US, such as 2-dimensional image and examiner dependency. Moreover, CT scan can provide information of the tumor status in neutral position; in contrast, the US probe could compress the patient's neck and may slightly distort the normal parenchyma around tumor. The thyroid gland is a compressible organ; therefore, external compression, by the probe of the US, can make the thyroid parenchyma thinner, especially when the thyroid is pressed between the malignant tumor and strap muscles. The distorted, thinner thyroid can misinform the physician about the actual proximity of the tumor and thyroid capsule. As shown in Figure 3, false positive findings occurring by US alone could be decreased if evaluated in combination with information from CT scans.

Regarding the ETE, CT scan showed plausible PPV comparing to US, especially when the tumor contacts with capsule more than 50%. However, there is still high chance of false negative and false positive prediction in CT scan. Combined interpretation of both US and CT was found to decrease the false negative and positive rates, thereby increasing the positive predictive value and AUC of ETE (Table 3). It was a higher value, compared with figures from other studies that evaluated ETE using US alone.

Despite the advantages of additional CT evaluation in predicting ETE, there are several limitations. First, very tiny nodule cannot be shown clearly in CT scan. Second, even in large tumors, some cases did not show clear margins and relationship with the thyroid capsule. In those cases, the categorization of ETE in CT scan could have a risk of arbitrary judgment and the physicians often had no choice but to solely depend on the US findings to predict the ETE. Third, CT is not used for routine preoperative workup, especially for predicting the ETE. Performing CT can be time and money consuming and also exposes patients to radiation. Therefore, we suggest that a thorough evaluation of thyroid gland using CT scan can make an additional contribution in predicting the ETE, in clinical settings where a preoperative CT scan is routinely performed, to evaluate for lymph node status and abnormality in anatomical structure.

In summary, this study suggests that CT scan can be a useful ancillary modality to predict the ETE when combining with US. The extent of surgery can be made based on the possibility of ETE. If normal parenchyma (possibly more than 1 mm in distance) surrounding the nodule can be demonstrated in both of US and CT scans, the possibility of ETE is very low and conservative surgical approach can be considered. In contrast, if the tumor has direct contact with the capsule in both of US or CT scans, ETE might be present in more than half of the tumors, especially in tumors larger than 1 cm. In this case wider extent of surgery might be considered. This could be decided by each surgeon according to the possibility of ETE as shown in Table 4. Further prospective study may elucidate the role of CT scan in predicting the ETE of thyroid cancer.

## 5. Conclusions

Although there is some limitation in CT scan to evaluate tiny thyroid nodule, additional information from the CT scan could increase the prediction of ETE with US. For those institutes using CT scan as routine examination preoperatively, thorough evaluation of the nodule might be helpful to determine the ETE.

## Figures and Tables

**Table 1:** Demographics and characteristics of patients.

	Without ETE (*n* = 203)	With ETE (*n* = 174)	All patients (*n* = 377)
Mean age	50.5 (±10.9)	47.6 (±10.2)	49.1 (±10.6)
Sex (M : F)	1 : 2.2	1 : 6.2	1 : 3.5
Mean size of nodule (cm)^*^	0.68 (±0.23)	1.19 (±0.36)	0.92 (±0.33)
Multicentricity	94 (43.6%)	99 (56.9%)	193 (51.2%)
Lymph node metastasis	9 (5.2%)	144 (83.7%)	153 (40.6%)

^*^ *P* < 0.001 (Fisher's exact test).

**Table 2:** Diagnostic accuracy of preoperative US and CT to predict the extrathyroidal extension.

	Group	Cutoff criteria	Sensitivity (%)	Specificity (%)	PPV (%)	NPV (%)	AUC (95% CIs)
US	Group 1						
		Contact with capsule	93.2	18.6	61.1	66.7	0.532 (0.415–0.666)
	Group 2						
		Disruption of capsule	66.1	65.1	72.2	58.3	0.647 (0.452–0.702)
	Group 3						

CT	Group A						
		Contact with capsule	86.8	30.2	52.4	72.2	0.483 (0.382–0.519)
	Group B						
		More than 25% contact	71.1	44.2	48.2	67.7	0.448 (0.335–0.491)
	Group C						
		More than 50% contact	23.7	95.4	81.8	58.6	0.638 (0.571–0.719)
	Group D						

US, ultrasonography; CT, computed tomography; PPV, positive predictive value; NPV, negative predictive value; CIs, confidence intervals.

**Table 3:** Diagnostic accuracy of combining the preoperative US and CT to predict the extrathyroidal extension.

Findings		Sensitivity (%)	Specificity (%)	PPV (%)	NPV (%)	AUC (95% CIs)
US	CT					
Contact with capsule	Contact with capsule	98.0	25.7	64.9	90.0	0.428 (0.352–0.574)
	More than 25% contact	87.8	48.6	70.5	73.9	0.472 (0.415–0.666)
	More than 50% contact	18.4	94.3	81.9	45.2	0.521 (0.483–0.637)

Disruption of capsule	Contact with capsule	97.6	40.7	71.9	91.7	0.654 (0.525–0.729)
	More than 25% contact	92.9	70.4	83.0	86.4	0.744 (0.646–0.802)
	More than 50% contact	59.5	92.6	92.7	59.5	0.725 (0.672–0.809)

US, ultrasonography; CT, computed tomography; PPV, positive predictive value; NPV, negative predictive value; CIs, confidence intervals.

**Table:** (a) Tumor less than 1 cm

Percentage of ETE+ (%)	CT features			
	Group A	Group B	Group C	Group D
US features				
Group 1	10.0	17.1	35.8	NA^*^
Group 2	22.5	45.2	42.1	**78.6**
Group 3	25.4	**62.9**	**87.0**	**92.7**

^*^NA: not applicable.

**Table:** (b) Tumor larger than 1 cm

Percentage of ETE+ (%)	CT features			
	Group A	Group B	Group C	Group D
US features				
Group 1	6.2	22.6	37.7	NA^*^
Group 2	13.6	**52.7**	**70.5**	**83.2**
Group 3	18.5	**75.4**	**89.1**	**94.5**

^*^NA: not applicable.
